# PancrESS – a meta-analysis resource for understanding cell-type specific expression in the human pancreas

**DOI:** 10.1186/s12864-024-09964-y

**Published:** 2024-01-18

**Authors:** David Sturgill, Li Wang, H. Efsun Arda

**Affiliations:** Laboratory of Receptor Biology and Gene Expression, Center for Cancer Research, National Cancer Institute, NIH, Bethesda, MD 20892 USA

**Keywords:** Pancreas, scRNA-Seq, Expression specificity, Meta-analysis

## Abstract

**Background:**

The human pancreas is composed of specialized cell types producing hormones and enzymes critical to human health. These specialized functions are the result of cell type-specific transcriptional programs which manifest in cell-specific gene expression. Understanding these programs is essential to developing therapies for pancreatic disorders. Transcription in the human pancreas has been widely studied by single-cell RNA technologies, however the diversity of protocols and analysis methods hinders their interpretability in the aggregate.

**Results:**

In this work, we perform a meta-analysis of pancreatic single-cell RNA sequencing data. We present a database for reference transcriptome abundances and cell-type specificity metrics. This database facilitates the identification and definition of marker genes within the pancreas. Additionally, we introduce a versatile tool which is freely available as an R package, and should permit integration into existing workflows. Our tool accepts count data files generated by widely-used single-cell gene expression platforms in their original format, eliminating an additional pre-formatting step. Although we designed it to calculate expression specificity of pancreas cell types, our tool is agnostic to the biological source of count data, extending its applicability to other biological systems.

**Conclusions:**

Our findings enhance the current understanding of expression specificity within the pancreas, surpassing previous work in terms of scope and detail. Furthermore, our database and tool enable researchers to perform similar calculations in diverse biological systems, expanding the applicability of marker gene identification and facilitating comparative analyses.

**Supplementary Information:**

The online version contains supplementary material available at 10.1186/s12864-024-09964-y.

## Background

The human body exhibits a remarkable diversity of cellular phenotypes, achieved through the selective deployment of the gene regulatory programs that result in specialized transcriptomes [1]. Within complex organs, these phenotypes enable individual cells to perform specialized functions crucial for organ homeostasis and function. Cell-type specific expression is achieved via multiple mechanisms and is evident in marker gene transcripts that differ in steady-state abundance between cell types. Identifying these targets is essential for understanding how expression is regulated. For example, imaging-based functional studies rely on marker genes to accurately identify cellular contexts based on this specialized transcriptional output. Reliable marker genes are critical for multiple research methods including fluorescence-activated cell sorting (FACS) [2], imaging via single-molecule FISH [3], and mass cytometry [4]. In pathology, marker genes serve as valuable tools for diagnostic purposes, allowing the identification and classification of different cell populations or disease subtypes based on their gene expression profiles. Furthermore, marker genes are essential for studying disease heterogeneity and understanding the cellular diversity within complex tissues [5]. For these reasons, multiple studies have sought to catalog cell-type specific expression in multiple organs, including the pancreas [6–8]. However, quantitative assessment of expression specificity within the major cell types of the pancreas, encompassing both endocrine and exocrine components, remains limited.

The pancreas holds significant clinical importance and carries a substantial disease burden globally [9, 10]. Understanding differential expression within pancreatic cell types would facilitate therapeutic discoveries, including generating insulin producing cells from stem cells for replacement therapies [5]. Indeed, identification of specific gene expression patterns in adult β-cells lead to the discovery of maturation factors now guiding stem cell approaches [11–14]. Additionally, diverse and reliable markers of cell identity are important for diagnostic purposes, particularly in diseased tissue where cell identities may be obscured [15].

Numerous transcriptomic studies have generated an abundance of data, including single-cell RNA sequencing (scRNA-Seq). However, scRNA-Seq technology has many variants, with multiple diverging protocols available over the past decade [16]. Each of these protocols has unique error profiles and systematic biases that may confound biologically relevant results when they are combined [16]. To maximize the utility of this disparate public catalog of data, they should be processed so that they are comparable and so that observed differences are biological rather than technical.

Despite the maturity of scRNA-Seq protocols, appropriate statistical methods remain under debate, with significant concern about false positive differential expression detection [17, 18]. In complex organs with multiple cell types, comparisons via multifactorial designs or multiple *inter-se* pairwise tests provide unclear pictures of differences. For these reasons, intuitive metrics expressing differences are a desirable complement to statistical testing.

In this work, we present a curated dataset of pancreatic scRNA-Seq data as well as a metric for relating the cell-type specificity of transcript abundances. We demonstrate that this approach accurately delineates previously characterized markers and identifies possible novel markers that can be applied to benefit in vivo studies of pancreatic disease. We describe our results as a reproducible method that can be applied to other biological contexts, to generally benefit any disease related experiments that utilize marker genes. To ensure generalizability, we offer software that calculates specificity metrics with customizable parameters. Taken together, we present a valuable resource for investigating cell type expression specificity in general and within the pancreas.

## Results

### Meta-analysis of pancreatic RNA-Seq data

**Fig. 1:**
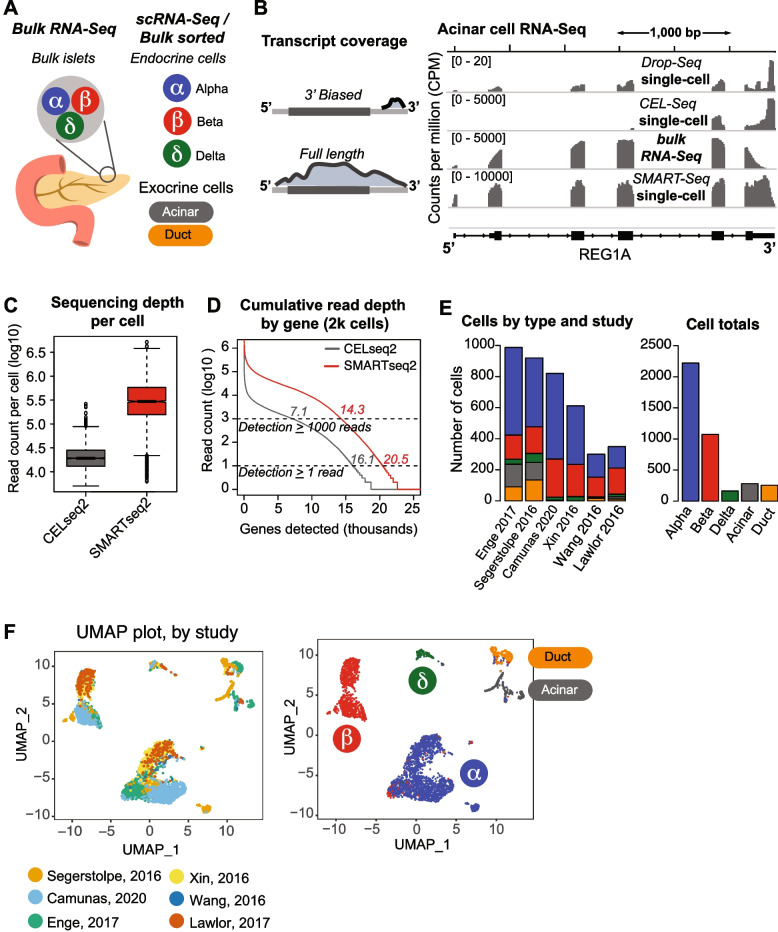
scRNA-Seq enables high-resolution interrogation of pancreatic cell-type specific expression. **A** Schematic of human pancreas and pancreatic cell types, contrasting bulk islet sequencing and single cell RNA sequencing. **B** Browser view RNA-Seq coverage of a representive acinar cell marker (REG1A). Tracks are depth normalized coverage tracks in units of counts per million (CPM). 3'-biased protocols (Drop-Seq/CEL-Seq) and full length (SMART-Seq) protocols are contrasted, as well as libraries from bulk, sorted cells and single cells. **C** Sequencing depth per cell in CEL-Seq (grey) and SMART-Seq (red) protocols. **D** Cumulative read count per gene in 2,000 CEL-Seq (grey) and SMARTseq2 (red) libraries. Numbers inset are the number of genes detected at a cumulative threshold of 1 read or 1,000 reads. **E** Number of cells in each study analyzed broken down by cell type (left) and the total number of cells by type. **F** Uniform Manifold Approximation and Projection (UMAP) of transcript abundances. At left: cells color coded by the study of origin, showing that data do not cluster by study. At right: cells color coded by cell type, showing that data cluster by cell type

The human pancreas is a compound organ consisting of two functional groups of cells: endocrine cells involved in hormone production regulating blood glucose levels (pancreatic islets) and exocrine cells involved in digestive enzyme secretion (Fig. 1A). Given the clinical importance of the pancreas, several studies using RNA sequencing have investigated differential expression between cell types. Many of these studies obscure cell-type specific expression within the pancreas by studying pooled bulk or bulk islets [19]. However, although several single-cell studies have been performed (Table S1), combining these data to arrive at a synthesis of cell-type specific transcriptional programs remains a challenge.

To leverage this large catalog of expression profiles and maximize their utility for understanding the biology of the pancreas, we sought to attain reference metrics of cell-type specific expression within pancreatic cell types. To calculate these metrics effectively, both depth and breadth of sequencing are critical – accurate pictures of the full complement of transcripts in an RNA sample as well as their relative abundances in each cell type are required. With this in mind, we evaluated data from the different types of protocols available, and found two major variations with respect to cell-type resolution and transcript coverage.

The biggest variation in protocol is with sample collection – specifically, the construction of libraries of whole islets or pancreas, bulk purified pancreas cells using flow cytometry (FACS) and individual cells using single-cell assays (Fig. 1A, B). Bulk collection with standard library preparation has an advantage over single-cell protocols in terms of total sequencing depth, but FACS by cell type is biased towards the availability of antibodies and cell surface antigens, and it is not trivial to remove signal from contaminating cell types *post-hoc*.

The other major protocol variation relates to resolution of the mRNA molecule that is assayed. While one group of protocols (CEL-Seq, Drop-Seq, 10X) captures 3’ ends of transcripts, the other (SMART-Seq) sequences the full transcript length (Fig. 1B) [20–22]. Full length detection affords several advantages for the identification of marker genes, such as better resolution of differential expression [23].

While 3’- biased protocols can also capture RNA abundances with single cell specificity, they suffer from reduced depth compared to SMART-Seq datasets. For example, representative CEL-Seq2 and SMART-Seq2 datasets show a ~ 15 × difference in median read depth per cell (Fig. 1C). Even when we restricted the read coverage to 3’ ends, we still find that CEL-Seq2 detects fewer transcripts, and cumulatively never reaches the same level of detection, either for a single-read or a robust level of read detection (1,000 reads) (Fig. 1D). This indicates significantly better breadth of gene detection for SMART-Seq. For these reasons, we compiled data from full-length SMART-Seq protocols, which gave us superior representation of the major pancreatic cell types when combined (Table S1, Fig. 1E). This dataset, comprising 4,695 cells derived from six studies [24–29], represents at least 150 cells of each major cell type. We recognize that there is substantial imbalance in representation of cell types, attributable to both the collection methodologies and the intrinsic cellular composition of the pancreas (Fig. 1E). However, a prior study demonstrated that correlations between single-cell data and bulk tissue measurements begin to plateau with the inclusion of only tens of single cells, indicating that even a relatively small number of cells can be sufficient to capture the expression profile of the tissue adequately [27].

To compare gene abundances across datasets, we implemented a simple consistent pipeline for read processing that implements two major routes of generating counts from RNA-Seq reads, alignment followed by annotation overlap, and k-mer based pseudoalignment via Kallisto [30] (Fig. S1A). After comparing counts from each path, we found gene level detection to be highly concordant (r >  = 0.92). For processing thousands of samples, the speed advantage of Kallisto made it the most practical choice for processing the entirety of the dataset. Following depth normalization into units of counts per million (CPM), results were visualized by dimensionality reduction via Uniform Manifold Approximation and Projection (UMAP). Highlighting clusters by study did not show extensive study-specific clustering, indicating the absence of strong batch effect (Fig. 1F). As expected, highlighting by cell type showed clear separation by cell-type specific transcriptional profiles, along with endocrine cells closer to each other than to exocrine cells (Fig. 1F). These results confirmed that with minimal normalization, meta-analyses of combined pancreatic scRNA-Seq results can effectively mitigate batch effects to identify biological differences. This also highlights that visual inspection of UMAP results is a useful check of batch effects when performing similar analysis in other contexts. Additionally, we recommend having sufficient representation of relevant cell types (ideally > 100 cells) to allow the resolution of potential batch effect problems.

### Expression specificity

The delineation of marker genes, representing the restriction of expression within a multicellular context, is commonly cited from early molecular studies or manually compiled from heterogenous sources [31]. Additionally, marker gene definitions are commonly categorical rather than quantitative. To express the concept of specificity in a formalized and bounded-scale manner, there have been multiple metrics proposed (reviewed in [32]). These metrics have similar motivations but have context-dependent performance differences. Many of the widely used metrics, such as Tau and the Gini coefficient, perform well at producing a single value for data across multiple tissues, but are not constructed to produce multiple values for each specific tissue or cell type [33]. For our goal of producing a specificity value for each expressed gene in each pancreatic cell type, we employed a metric which we call the Expression Specificity Score (ESS), to assess the degree to which transcription is restricted to certain cells, which was defined in a previous study of gene expression in the pancreas, although on bulk, FACS purified cells [34]. In this work, we adapt this metric to accommodate the integration of multiple single-cell transcriptomic data.

**Fig. 2:**
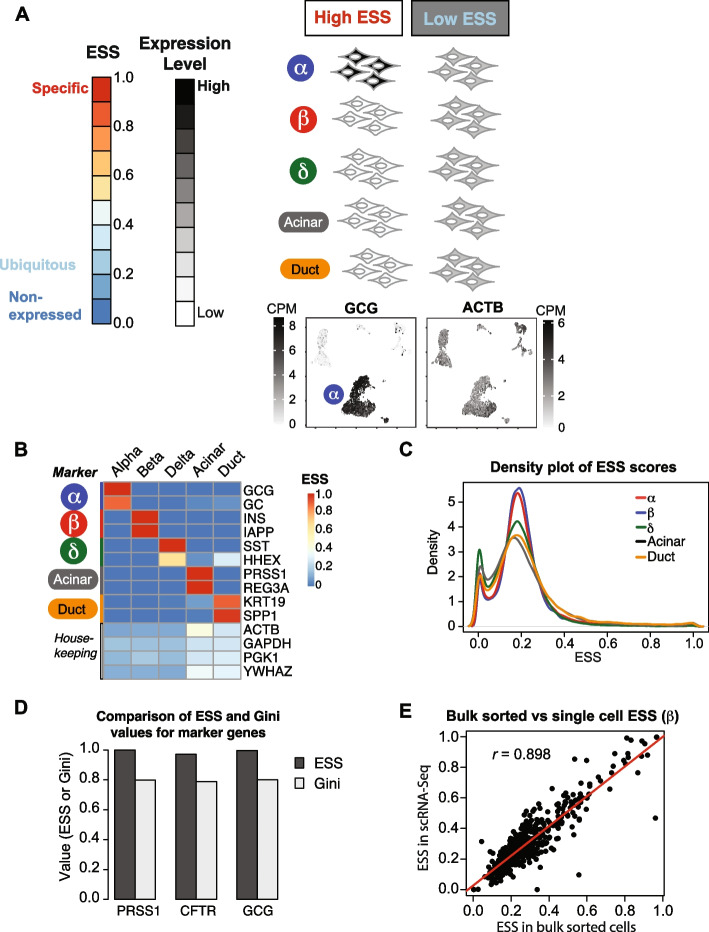
Expression Specificity Score (ESS) quantifies cell-type specific expression. **A** Schematic of the ESS scale and its interpretation in the context of pancreatic expression levels. Cartoons of cells represent distinct pancreatic cell-types, grey shading indicates expression levels of glucagon (*GCG*) or actin (*ACTB*) in these cell-types. UMAP plots of α-cell specific expression of glucagon gene (*GCG*) and the housekeeping gene actin (*ACTB*) are shown. The latter UMAP shows moderate expression of this gene in all cell types. **B** Heatmap of ESS values for representative known markers for each cell type, along with housekeeping genes, to demonstrate the concordance of ESS with expected values in known targets. **C** Density plot of ESS values for all genes in each cell type. **D** Bar chart of specificity scores for known markers using the ESS and Gini metrics. **E** Scatter plot comparing ESS in scRNA-Seq to ESS in bulk sorted beta cells, calculated using α-, β-, acinar, and duct cell data. Results for the top expressed 500 genes in beta cells are shown

We used our compiled meta-analysis of scRNA-Seq data to quantify ESS by cell type within the normal human pancreas. Briefly, the ESS calculation takes a summary measure of gene level abundance in each cell type, and divides by the sum of these measures across cell types (Fig. S1B, see *Methods*). This produces an intuitive metric bounded by 0 and 1 that reflects the restriction of expression to each single cell type (Fig. 2A). Taking the maximum ESS across cell types represents the general specificity within the pancreatic context. High cell-type specific expression, such as α-cell specific glucagon gene, produce values close to 1 within α-cells, while constitutive housekeeping expression produces values around 1/N in each cell type, where N is the number of cell-types (or 0.20 in our case with five cell types) (Fig. 2A). Through this unsupervised approach, the ESS reflects expected values in known cell-type markers in each cell type, as well as within housekeeping genes (Fig. 2B).

Although high ESS genes are generally highly expressed in their relevant cell type, this metric also captures the specificity of low and moderately expressed genes, given adequate sampling depth. Additionally, in scRNA-Seq data it allows for detection of specificity for target genes that are not uniformly detected in single cell libraries. For example, the gene *PTF1A* is an important acinar cell fate regulator that is not highly expressed yet has an ESS of 0.98 in acinar cells (Fig. S1C).

The ESS calculation is similar to the tissue specificity index (TSI) [32], with a variation that allows the calculation of a score in each cell or tissue type. As constructed, the ESS has flexibility in how it summarizes available data, so that it can be varied according to the applied dataset. The two major decisions in calculating ESS are the method of gene-level summarization (e.g. median or mean), and the method of aggregation (by cell type or study) (Fig. S1B). In this study, performing library size normalization (via DESeq2) after summing by cell type within each study, followed by taking medians within each cell type, provided the highest sensitivity for gene detection while mitigating cell count imbalances between studies (Fig. 2B, Table S2). ESS is calculated so that each gene has a reported value in each cell type. A general value for the tissue or organ of interest consists of the maximum value among the composite cell types. Choosing this method, we visualized the distribution of ESS in density plots for each cell type as a useful way to compare distributions between cell types [32]. From this we see that high cell-type specificity is the exception and not the rule, with the majority of genes expressed across cell types (Fig. 2C).

To compare our ESS with other metrics, we built into our tool functions to calculate them, including Gini [33, 35] and Tau [32]. When comparing results between these metrics, both ESS and Tau provide comparable high values close to one for specificity. The Gini coefficient however, since it is constructed to calculate within larger populations, fails to provide a value close to one when calculating within the set of pancreatic cells we analyzed (Fig. 2D). To compare our results in scRNA-Seq to bulk sorted cells, we calculated each consistently in a four-cell type fashion (since bulk sorted delta cell data is not available). This showed that ESS is robust to this technical difference – scores were largely concordant with average Pearson correlation coefficients of 0.866 in acinar, 0.7238 in duct, 0.8529 in α-, and 0.8979 in β-cells (Fig. 2E). The lowest correlation was in duct cells, which has the lowest representation of cells in our dataset. This illustrates that higher sample number improves ESS consistency.

One technical challenge of scRNA-Seq experiments, particularly for the pancreas due to containing cell types that produce large amounts of secreted peptides, is contamination from exogenous RNA. For instance, insulin mRNA produced by β-cells can contaminate the library of another cell type on the same plate via fluidic carryover. This kind of contamination was recently observed in GTEx samples in libraries of other tissues processed on the same day as pancreatic samples [36]. We also observe a small amount of outlier insulin transcript detected in non-β-cell types, including high CPM values in α- and δ-cells (Fig. S1D). Our ESS approach helps to mitigate this pernicious problem. By using medians (or other metrics of summarization), contaminating signal is effectively diluted or nullified, such that the calculated ESS value reflects the true specificity of expression. In this example, the calculated ESS is close to 1 in β-cells (0.996) and close to 0 in other cells (< 0.0016) (Table S2).

### Online resource and ESS query interface

Examining the genes with high specificity reveals an expanded set of putative pancreatic cell markers: 939 genes with high ESS of 0.8 or higher (Table S2), providing a resource of additional options for study design that involves marker genes. We also compiled previously published marker gene definitions, to indicate where these definitions may be in different contexts (eg, within islet cells only, see Table S3). We identified novel marker genes, including 513 protein coding genes and 133 non-coding transcripts (Table S3).

**Fig. 3:**
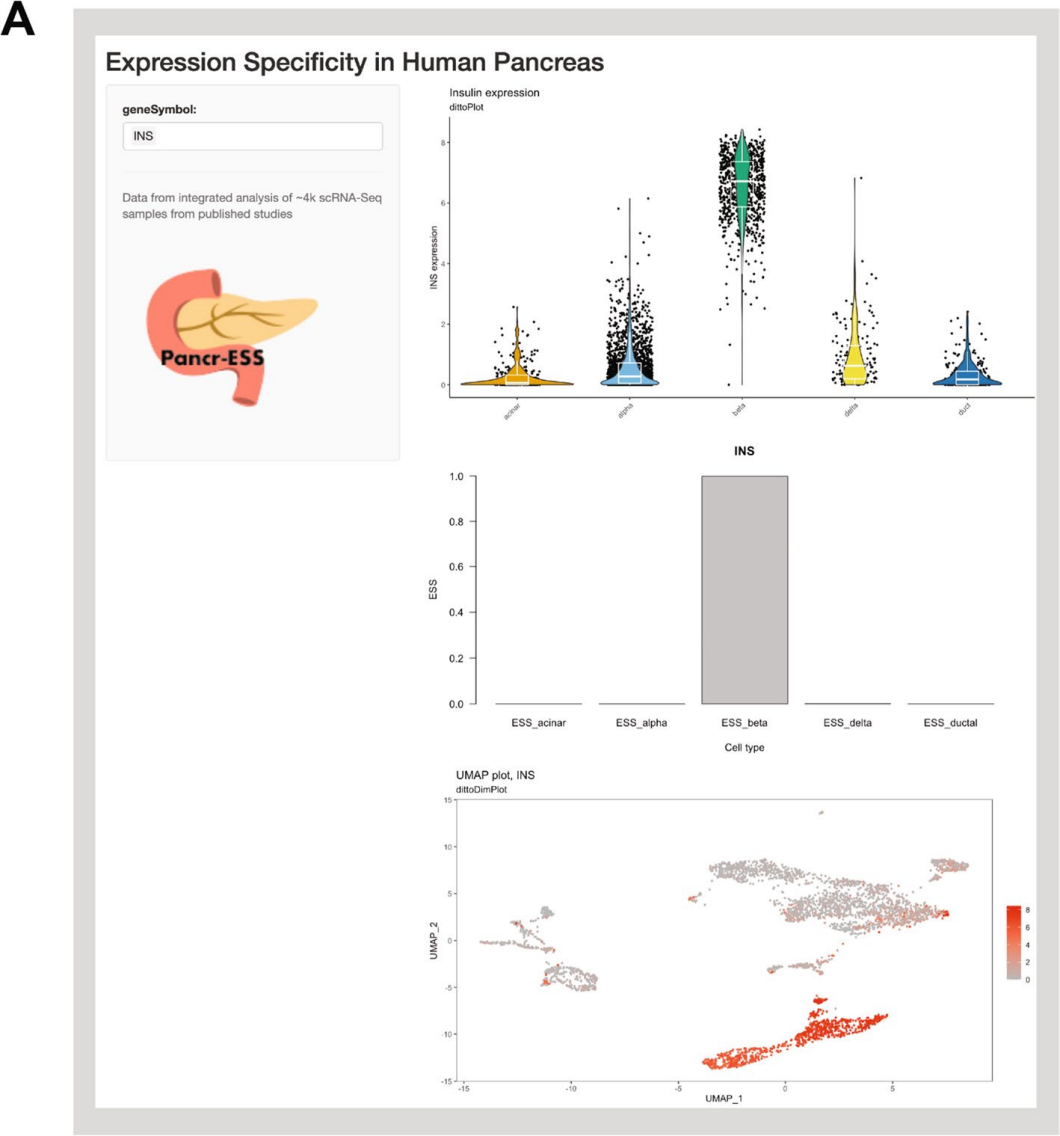
The pancrESS database interface. **A** Screen capture of the search interface for pancrESS. Users can select a gene symbol (left) and obtain a UMAP representation of combined data with the gene of interest highlighted, along with detailed ESS and transcript abundance levels

To facilitate the use of this work as a reference transcript abundance and specificity resource, we designed an online tool (Fig. 3). This resource takes gene symbols as input, and returns the relevant expression levels and ESS. The user may also select options including the gene expression aggregation method, transcript abundance metric, and cell-type context. Tabulated data along with several visualizations are produced, include boxplots and a UMAP representation of expression levels. The latter is useful as a concrete example of the specificity as well as a visualization of the heterogeneity of expression.

In addition to the catalog of ESS data, we generated reproducible code to calculate ESS along with other associated statistical metrics, like the Gini coefficient [37] or Tau [38]. With this code, ESS metrics can continually be recalculated when additional data are generated, or applied to data from other organ systems. This code is available at https://github.com/dsturg/PancrESS

## Discussion

In this work, we performed a meta-analysis of pancreatic single-cell RNA-seq to quantify cell-type specific gene expression. We observed a substantial sensitivity advantage of data from SMART-Seq2 libraries over 3’ biased protocols, consistent with other studies [20, 21], which was critical to the generation of complete transcriptional profiles. Although our analysis obtained adequate transcript coverage, we note that protocol improvements are available which may perform better for the generation of new data. For example, SMART-Seq3 [39] and G&T-seq [40] have recently been shown to perform best for transcript detection sensitivity [41].

We described and implemented an expression metric, the Expression Specificity Score (ESS), which allows the quantification of gene expression specificity in each pancreatic cell type. The ESS stands out for its intuitive and bounded metric system, allowing researchers to gain insight into the extent of restriction of gene expression within a single cell type. A significant technical challenge we navigated is the potential contamination from ambient RNA, particularly relevant for the pancreas, where cell types often produce large quantities of secreted peptides. A few groups developed computational approaches to remove or correct the contaminating transcripts from scRNA-Seq datasets [42–44]. While these studies address an important issue in single cell analysis, they can sometimes limit the detection of cell-to-cell variability or marker genes. Our ESS metric offers a complementary approach– we demonstrated that by employing medians or other summarization metrics, we effectively dilute or eliminate the contaminating signal, thus revealing the genuine specificity of expression. Furthermore, ESS can capture the specificity of genes across the expression spectrum, from low to high, improving on the identification of target genes which may be undetected in single cell libraries.

We would like to acknowledge that due to insufficient data, we were not able to include minor cell types of the pancreas, like pancreatic polypeptide cells (PP cells), epsilon cells, or pancreatic stellate cells. These cell types were included in some studies [26, 27], but with sample sizes too low to enable equal consideration. We note that in a previous meta-analysis, PP cells were included from these studies, but this analysis was restricted to islet cells [8]. Despite this challenge, our approach maintains flexibility and is adaptable to additional cell types. As more data become available, our methodology allows for an easy integration of these minor cell types, further enhancing the comprehensiveness and usefulness of the ESS.

Our database and analytical tool can be extended to other biological systems, which significantly broadens their applicability. These tools are expected to be a valuable resource for the scientific community and facilitate comparative analyses across different cell types or biological systems.

## Conclusions

In summary, we have described a catalog of expression profiles of pancreatic endocrine and exocrine cell types, and a resource for specificity metrics within the pancreatic system. The latter will serve as a valuable tool for marker gene identification, which are critical for increasingly complex in vivo experiments. Beyond the pancreatic system, ESS provides a flexible framework for application to different datasets that may be different in terms of breadth and depth. When combined with other disparate omics experiments to make connections between different regulatory mechanisms at play, our approach provides a discrete score to facilitate integration [45, 46].

## Methods

### scRNA-Seq protocol comparison

For the RNA-Seq protocol comparison presented in Fig. 1, representative acinar samples were aligned to the GRCh38 assembly using STAR [47] and visualized with the IGV browser (Broad Institute, [48]). Samples shown are: Drop-Seq (SRR5818089, GSM2700339 [49]), CEL-Seq (SRR4003812, GSM2262817 [19]), bulk RNA-Seq (SRR1299333, GSM1398975, [11], and SMART-Seq2 (ERR1630022, [27]). Samples were selected as representative of the depth obtained per sample in their respective experiments. We note that this selection broadly illustrates protocol differences, but is not meant to reflect a canonical acinar cell result, which in fact may be highly heterogeneous [50].

To explore detection ability differences by protocol, we analyzed pre-processed pancreas scRNA-Seq data from Satija et al., packaged as part of Seurat v.3 [51]. This dataset is also described in the instruction page at: https://satijalab.org/seurat/archive/v3.0/integration.html

This dataset is an independently compiled and well described standardized dataset, that aims to integrate data from multiple platforms. Thus, it serves to help isolate the effect of depth from other analysis parameters. This analysis views the effects of depth distinctly, independently of our downstream analysis pipeline. Protocols were compared visually in a genome browser, to assess coverage over marker gene transcripts. Additionally, depth per cell and gene detection was assessed, using precomputed counts from the source described above. For the former, we generated boxplots of read counts per cell and compared distributions. For the latter, we used an R script to calculate cumulative sums of genes detected at different total read counts, and indicated results at thresholds of 1 or 1,000 reads.

### RNA-Seq processing

#### Data acquisition

Pancreatic single-cell RNA-Seq studies were identified from the literature and data repositories (PRISMA flow diagram, Fig. S2). Briefly, following our evaluation of scRNA-Seq data by protocol, we proceeded to compile single-cell pancreatic RNA-Seq data that used the SMART-Seq protocol, via literature searches in Pubmed and keyword searches in the Gene Expression Omnibus (GEO). Within identified studies, we selected those that included at least 100 samples, from normal adult (rather than pediatric or disease samples). RNA-Seq data were then obtained from public repositories, via accessions listed in Table S1. Data for bulk sorted cells for comparison were processed in equivalent fashion and acquired from accession GSE79469 [11]. Where necessary, reads were trimmed of low quality base calls and adapter contamination using cutadapt [52].

All analysis used the GRCh38 assembly, with transcript abundances quantified against RefSeq annotation (NCBI Homo sapiens Updated Annotation Release 109.20191205).

#### Quality control

Exploratory alignments to measure contamination and sequencing artifacts were performed with the Bowtie2 aligner (v.2.4.1) [53], specifying “–sensitive-local" alignment parameters, to relevant contaminant reference sequences. Quantification of rRNA was performed by alignment with to the 43 kb Human ribosomal DNA complete repeating unit (U13369.1) downloaded from GenBank. Alignment fractions to rRNA in tested samples were low. To test the impact of in-silico rRNA read removal on downstream results, we extracted the rRNA unaligned reads from a sample, and compared Kallisto transcript abundances with and without this separation. These results demonstrated no impact of in-silico rRNA removal, so we did not perform this processing step on all samples for downstream analysis. To spot check for other contaminants in each dataset, the Sponge database was used [54] From these results, no significant contaminant that would affect biological integrity of samples (e.g.; mycoplasma) was detected.

#### Processing pipeline

Transcript counts for published RNA-Seq data were obtained from two standardized methods, to enable comparison between experiments. These pipelines were chosen to represent the major approaches used in the literature (Fig. S1A). The first pipeline is representative of the alignment based approach, where raw reads are first aligned to a genomic reference, and read overlap with coordinates of gene features is quantified. This pipeline uses the STAR aligner v.2.7.3a [47] and featureCounts (Subread v.2.0.1) [55]. The second pipeline is representative of the alignment independent approach, where transcripts are quantified using the sequence content of reads, using a pre-built index of k-mer content of transcripts. This pipeline uses Kallisto v.0.46.1 [30]. Transcript abundances were compiled and summarized at the gene level using Tximport [56].

To confirm the strandedness of the library preparation protocol, we used the *infer_experiment.py* script within the Rseqc tool [57]. From this result, we set downstream abundance calculation parameters accordingly.

#### Parameter specification

From the normalized Seurat object described in the meta-analysis below, we used the FindVariableFeatures Seurat function with the “vst” selection method and 2000 features. We then scaled the data using the ScaleData function. Principle component analysis (PCA) was run using RunPCA and the defined variable features. Clustering was performed using FindNeighbors and dims 1:10, followed by FindClusters, specifying 0.5 for resolution. The UMAP was generated using Seurat’s runUMAP function. These parameters generally followed default Seurat vignettes, with number of samples and features selected based on compute time performance.

### Cross-experiment meta-analysis

#### Filtering criteria

Prior to normalization and meta-analysis, filtering is performed to remove low-quality (single cell) libraries. The filtering criteria were a minimum count numbers in the housekeeping gene ACTB (minimum 100 reads) as well as a minimum total count (minimum 250,000 total reads). These thresholds were selected from the distributions of counts across cells, and represented the lowest 5% and 10% of values, respectively. Enforcing the dual cutoff preserved 89% of cells.

#### Normalization strategies

To combine gene abundance estimates across experiments, we normalized values in each cell to make them comparable in the combined data via one or more strategies. In the simple counts per million (CPM) normalization, counts for each gene in each cell are divided by the sum of counts across genes in that cell (in millions). The analogous normalization is performed on the pseudobulk pooled variation. The advantage of this normalization method for exploratory analysis of gene level abundance is that it is conceptually simple, allowing for comparison between cells and experiments normalized for depth, without potential ambiguity from normalization by transcript length. Expression results in units of transcripts per million (TPM), which do account for transcript length, are provided in Table S2. For the ESS results presented in Table S2, raw counts in individual cells of the same type are summed by experiment, followed by size-factor normalization with DESeq2 [58]. The rationale for this approach is to better balance different sized experiments, and reduce zero-inflation of low expressed genes. ESS values calculated with these normalized values demonstrated high specificity scores of known markers as well as the absence of skewed distributions in each cell type. In UMAP representations where input data were raw counts, normalization was performed using the Seurat normalizeData function (v.4.0) [51].

### Clustering and UMAPs

To assess distance between cellular transcriptome profiles, we performed dimensionality reduction using Universal Manifold Approximation and Projection (UMAP). This was implemented in the Seurat package (v. 4.3.0.1) [59], with graphical improvements from the dittoSeq package (v.1.12.2) [60].

### Compilation of previously published marker gene definitions

We compiled lists of previously defined marker genes from the literature (Table S3), via curation of original sources and compilation databases [8, 19, 31, 61]. These sources were identified via literature search, by reviewing literature cited in the primary studies we used for RNA-Seq data, with additional searches using “pancreas” and “marker” keywords. For each source identified, we compiled marker definitions where at least three islet cells were represented with at least ten genes. No additional restrictions were applied, and gene lists were manually re-typed when tabular format was not available.

### Expression specificity score

To examine the cell-type specificity of gene expression, we generated the expression specificity score (ESS). Several variants of this calculation were compared for evaluation, using formulae compiled from Kryuchkova-Mostacci et al*.* [32]. Additional information on the calculation is presented in the results section, with schematics in Fig. 2A and Fig. S1B. Criteria for evaluation included visual inspection of CDF distributions as in Kryuchkova-Mostacci et al*.*, high ESS values for a subset of known markers, and low ESS values for housekeeping genes (Fig. 2C). The edgeR Bioconductor package was used to calculate Gini values [35].

Functional annotation enrichment analysis (Fig. S3) was performed by entering gene lists of high ESS genes into the EnrichR server (https://maayanlab.cloud/Enrichr/) (Chen et al., 2013). Pathway annotation enrichment analyses were performed within the server.

### Browser views and visualization

To produce depth-normalized coverage tracks from RNA-Seq data, the deepTools package [62] was used, selecting a bin size of 25 bp and the Coverage Per Million (CPM) metric. We chose this tool because it is open source with a peer reviewed publication, and includes flexible parameters for binning and normalization. For these parameters, we chose a bin size of 25 bp to balance file size and resolution, and CPM normalization to equilibrate depth, Genome browser views for visualizing read density were generated using the Integrated Genomic Viewer (IGV) browser (Broad Institute, [48]), which has memory-efficient performance with multiple tracks, and allows export in an editable format.

### Web server and code availability

The online resource we developed for ESS scores and dynamically generated plots is linked from the project page at https://github.com/dsturg/PancrESS. This site was built using Shiny, which facilitates lightweight web implementations of R programs [63]. Reproducible code that takes gene level measurements as input, and generates our ESS or other specified specificity metrics, is also available at https://github.com/dsturg/PancrESS. We invite the community to provide feedback, fork, and contribute to the development of this resource via this repository.

### Supplementary Information

**Additional file 1: Figure S1.** RNA-Seq processing and ESS details. (A) Schematic of scRNA-Seq primary quantitation pipeline. (B) Schematic of ESS calculation steps with variations. (C) UMAP plot showing acinar specific detection in a low-expressed gene. (D) Insulin transcript detection in each cell type. Outlier spots show detection of exogenous RNA.** Figure S2.** PRISMA flow diagram for study selection.** Figure S3.** Functional annotation enrichment of high ESS genes. Functional enrichment results from the EnrichR tool (Chen et al., BMC Bioinformatics 2013), from the Ma’ayan Lab (https://maayanlab.cloud/Enrichr).

**Additional file 2: Table S1.** RNA-Seq datasets.

**Additional file 3: Table S2.** Expression specificity scores.

**Additional file 4: Table S3.** Pancreatic marker gene compilation.

## Data Availability

Computed ESS scores and visualizations, along with reproducible code for ESS calculation is available at: https://github.com/dsturg/PancrESS.
