# Supplementary material for: PancrESS – a meta-analysis resource for understanding cell-type specific expression in the human pancreas
Source: BMC Genomics. 2024 Jan 18;25:76. doi: 10.1186/s12864-024-09964-y (PMC10797729; doi:10.1186/s12864-024-09964-y)
Supplement: Supplementary file 1 — Additional file 1: Figure S1. RNA-Seq processing and ESS details. (A) Schematic of scRNA-Seq primary quantitation pipeline. (B) Schematic of ESS calculation steps with variations. (C) UMAP plot showing acinar specific detection in a low-expressed gene. (D) Insulin transcript detection in each cell type. Outlier spots show detection of exogenous RNA. Figure S2. PRISMA flow diagram for study selection. Figure S3. Functional annotation enrichment of high ESS genes. Functional enrichment results from the EnrichR tool (Chen et al., BMC Bioinformatics 2013), from the Ma’ayan Lab (https://maayanlab.cloud/Enrichr). [file 12864_2024_9964_MOESM1_ESM.docx]

**Sturgill et al., Supplementary Figure S1**

**
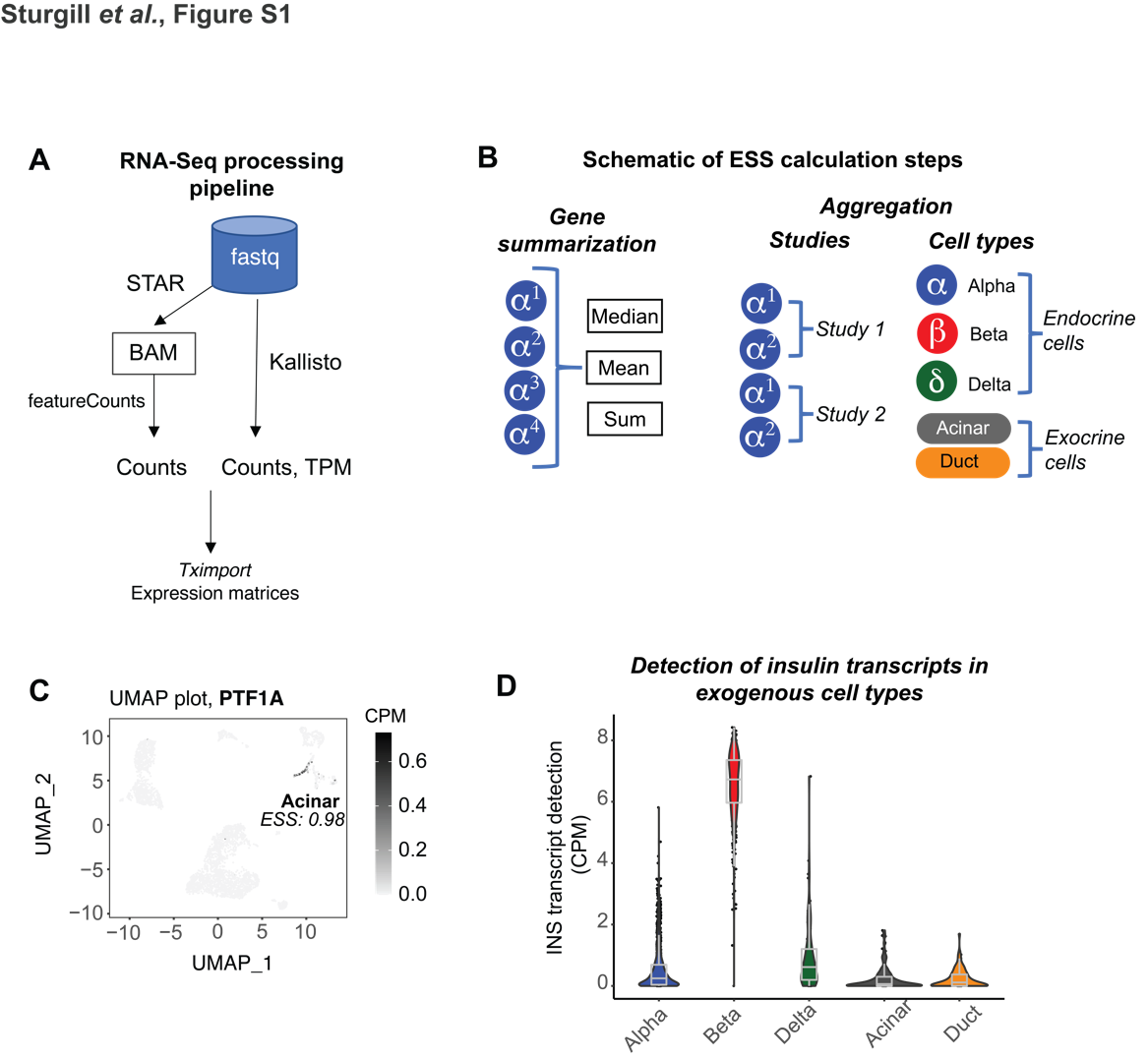
**

**Figure S1: RNA-Seq processing and ESS details.** (**A**) Schematic of scRNA-Seq primary quantitation pipeline. (**B**) Schematic of ESS calculation steps with variations. (**C**) UMAP plot showing acinar specific detection in a low-expressed gene. (**D**) Insulin transcript detection in each cell type. Outlier spots show detection of exogenous RNA

**Sturgill et al., Supplementary Figure S2**

Sturgill *et al.*, Figure S2

**Identification of studies via databases and repositories**

Articles identified from Pubmed searching

(n = 128)

Data submissions identified on GEO

(n = 203)

**Identification**

Studies identified (>100 normal single cell samples)

(n = 9)

**Screening**

Studies excluded

(n = 3 )

Studies performed with full length protocols

(n = 6)

Studies included in review

(n = 6)

**Included**

**Figure S2: PRISMA flow diagram for study selection**

**Sturgill et al., Supplementary Figure S3**

Sturgill *et al.*, Figure S3


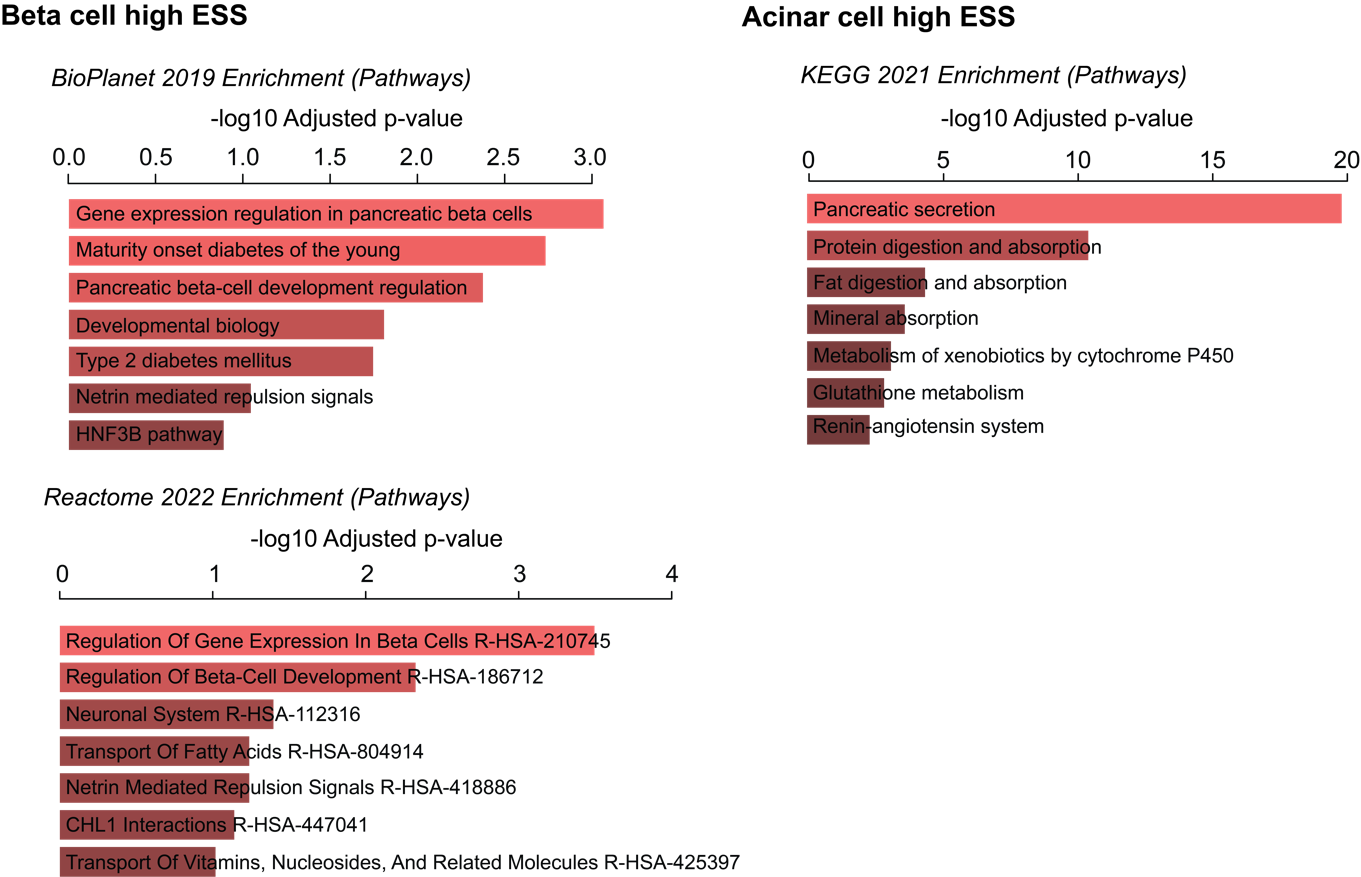


**Figure S3: Functional annotation enrichment of high ESS genes.** Functional enrichment results from the EnrichR tool (Chen et al., BMC Bioinformatics 2013), from the Ma’ayan Lab (https://maayanlab.cloud/Enrichr).
